# Habitual FODMAP Intake in Relation to Symptom Severity and Pattern in Patients with Irritable Bowel Syndrome

**DOI:** 10.3390/nu13010027

**Published:** 2020-12-23

**Authors:** Sanna Nybacka, Stine Störsrud, Helen M. Lindqvist, Hans Törnblom, Magnus Simrén, Anna Winkvist

**Affiliations:** 1 Institute of Medicine, Sahlgrenska Academy, University of Gothenburg, Box 459, 405 30 Göteborg, Sweden; stine.storsrud@vgregion.se (S.S.); helen.lindqvist@gu.se (H.M.L.); hans.tornblom@gu.se (H.T.); magnus.simren@medicine.gu.se (M.S.); anna.winkvist@nutrition.gu.se (A.W.); 2 Center for Functional Gastrointestinal and Motility Disorders, University of North Carolina at Chapel Hill, Chapel Hill, NC 27599-7080, USA

**Keywords:** irritable bowel syndrome, FODMAP, IBS symptom severity, diet, IBS subtypes

## Abstract

Restricting intake of FODMAPs (Fermentable Oligo-, Di-, Monosaccharides and Polyols) is used as treatment for irritable bowel syndrome (IBS). However, whether habitual FODMAP consumption correlates to symptom severity, and if this relationship differs among IBS subtypes, is unclear. The aim was to study the relationship between habitual FODMAP intake and symptom severity. A total of 189 patients with IBS—IBS with constipation (IBS-C) *n* = 44 (22.3%), IBS with diarrhea (IBS-D) *n* = 54 (27.4%), mixed IBS (IBS-M) *n* = 46 (23.4%) and unsubtyped IBS (IBS-U) *n* = 46 (23.4%)—recorded food intake during four days. Symptom severity was measured with the IBS severity scoring system (IBS-SSS). For FODMAP intake, a lower lactose intake was noted among women with IBS-D, *p* = 0.009. In women, there was a statistically significant relationship between energy-adjusted FODMAP intake and IBS-SSS (r = 0.21, *p* = 0.003). This was mainly driven by the subtype IBS-U, where excess fructose intake accounted for 19.9% of explained variance in IBS-SSS (*p* = 0.007). This study demonstrates small differences in FODMAP intake among IBS patients with different subtypes. Association between IBS symptoms and FODMAP intake was most prominent in unsubtyped IBS. However, patients who are intolerant to certain FODMAPs may already have reduced their FODMAP intake, and this warrants future cohort or experimental studies to uncover.

## 1. Introduction

Irritable bowel syndrome (IBS) is a common gastrointestinal (GI) disorder that is characterized by recurrent abdominal pain together with an abnormal frequency and/or consistency of stools [1]. The diagnosis is based on fulfilling the Rome criteria for IBS without any signs of an organic GI disease on limited diagnostic testing [1]. Although IBS is associated with a large health care utilization [2,3], efficient treatment options are still limited. As many patients with IBS report diet as a major factor in triggering or worsening GI symptoms [4,5,6,7], dietary treatment is often considered as a first-line option. There is some evidence that intake of alcohol [8,9], caffeine [5,8], and spicy foods [4,8], as well as foods rich in fat and carbohydrates [4,5] might trigger symptoms and should thus be limited in intake, but not all patients respond to these dietary modifications. Patients with IBS differ from each other in symptom patterns and severity and have different underlying pathophysiology; there is still much to be done in developing dietary therapies that target different subtypes of IBS and with different underlying mechanisms of the disease.

Restricting intake of fermentable carbohydrates, FODMAPs (Fermentable Oligo-, Di-, Monosaccharides and Polyols) is increasingly recommended in the management of IBS. The low-FODMAP diet focuses on reducing intake of poorly digestible carbohydrates, such as galacto-oligosaccharides (GOS), fructans, lactose, fructose, and sorbitol [10]. These carbohydrates are typically found in wheat-based products, onion, garlic, legumes, dairy, and a wide range of fruits and vegetables. The common trait among these carbohydrates is that they are incompletely absorbed in the small intestine, which can cause gas production due to rapid fermentation and to an osmotic action with increased water retention. Thus, reducing FODMAP intake could lead to less luminal distention and, thereby, less pain [10]. However, as some FODMAPs also act as prebiotics [11], a reduction in FODMAPs will concurrently reduce the prebiotic intake, and concerns have been raised if this will affect the gut microbiota in an unfavorable manner in the long term [12].

To date, most dietary studies on FODMAP intake in patients with IBS have been focusing on restricting or limiting intake of all FODMAPs during a period of time, followed by a reintroduction of selected FODMAPs and evaluating the change in symptom severity and patterns. As a short-term dietary treatment, the low-FODMAP diet seems effective in reducing GI symptoms in around 50–80% of the treated patients in randomized controlled trials [13,14,15,16]. In a previous study, we have reported that the habitual intake of FODMAPs varies largely between individuals with IBS, but there is also a large day-to-day variation within individuals [17]. Little is still known about how the habitual FODMAP intake correlates to symptom generation and severity in this patient group, and if amounts, types, or intake patterns matter. In addition, not much is described regarding whether this relationship differs among IBS subtypes. The aim of this study was to evaluate the relationship between FODMAP intake and symptom severity and pattern in patients with IBS, taking advantage of existing detailed dietary intake data among well-characterized patients of different IBS subtypes.

## 2. Materials and Methods

### 2.1. Study Design

This study is a secondary analysis taking advantage of detailed dietary and clinical data assembled at baseline within two clinical studies performed at our specialized unit for functional GI disorders at the Sahlgrenska University Hospital, Gothenburg, Sweden, between 2011 and 2014. The study population has been described in detail before [14,18]. Both studies were registered at www.clinicaltrials.org; NCT02107625 and NCT01252550. All subjects gave their informed consent for inclusion before they participated in the studies. The studies were conducted in accordance with the Declaration of Helsinki, and the protocols were approved by the Ethics Committee of Gothenburg (no. 613-19 and 731-09). 

The inclusion criteria were women and men ≥18 years of age diagnosed with IBS according to the ROME III criteria. Patients were excluded if they had other GI diseases explaining their symptoms, other serious chronic diseases, severe psychiatric diseases, alcohol abuse, were taking probiotic supplements, were pregnant, had abnormal results on standard screening laboratory tests, or if they were unable to reliably respond to questionnaires in Swedish. In one of the trials, only subjects with moderate to severe symptoms (i.e., IBS severity scoring system (IBS-SSS) ≥ 175) were eligible for enrolment. At baseline visit, the IBS diagnosis was confirmed by a physician according to the Rome III criteria [1]. To facilitate with IBS subtyping, participants filled in a 10-day stool diary using the Bristol stool form chart (BSF). Subtypes of IBS are divided into four categories based on the predominant stool form; IBS with predominant constipation (IBS-C, ≥25% hard stools (BSF 1 or 2) and <25% loose stools (BSF 6 or 7)), IBS with predominant diarrhea (IBS-D, ≥25% loose stools and <25% hard stools), IBS with alteration between constipation and diarrhea (IBS-M, ≥25% of reported stools hard and ≥25% loose); and unsubtyped IBS (IBS-U, insufficient abnormality of stool consistency to meet criteria for IBS-C, -D, or -M). Moreover, patients’ demographics were assessed and the patients completed questionnaires, see below.

### 2.2. Questionnaires

#### 2.2.1. IBS Severity Scoring System (IBS-SSS)

The IBS severity scoring system (IBS-SSS) is a questionnaire commonly used to evaluate the severity of IBS symptoms [19]. Five questions are included in an overall IBS severity score: abdominal pain intensity, abdominal distension, bowel habit dissatisfaction, and life interference, each rated with visual analogue scales (0–100 each), and abdominal pain frequency, which is defined as the number of days with pain during the last 10 days multiplied by 10 (0–100). This leads to a total score between 0 and 500, with higher scores indication more severe symptoms. Patients with IBS-SSS < 175 are defined as mild IBS, 175–300 as moderate IBS, and >300 as severe IBS [19].

#### 2.2.2. The Patient Health Questionnaire (PHQ-15)

The Patient Health Questionnaire (PHQ-15) is a questionnaire used for screening for somatization and monitoring somatic symptom severity [20]. The questionnaire inquires 15 different somatic symptoms or symptom clusters (mental, physical, social, general, and pain) that account for more than 90% of the physical complaints (excluding upper respiratory tract symptoms) reported in the outpatient setting. Each symptom is scored from 0 (“not bothered at all”) to 2 (“bothered a lot”).

#### 2.2.3. Hospital Anxiety and Depression (HAD) scale

The Hospital Anxiety and Depression (HAD) scale is an instrument for screening the severity of clinically significant symptoms of anxiety and depression in patients [21]. The scores are not affected by bodily illness, i.e., symptoms that have arisen from somatic and/or mental disorders are excluded. The scale consists of 14 items, each using a 4-point Likert scale (0–3), with subscales for anxiety (7 items) and depression (7 items), which provides a score between 0 and 21 on each subscale. The higher the score, the more prominent are the symptoms. 

#### 2.2.4. Visceral Sensitivity Index (VSI)

The VSI questionnaire measures GI-specific anxiety, and it includes the cognitive, affective, and behavioral response to fear of GI symptoms, and the context in which these occurs [22]. The questionnaire contains 15 questions, each scored between 0 and 5, giving a total maximum score between 0 and 75. A higher score indicates a more severe GI-specific anxiety.

#### 2.2.5. Multidimensional Fatigue Inventory-20 (MFI-20)

The Multidimensional Fatigue Inventory-20 (MFI-20) assesses the severity of general fatigue, physical fatigue, reduced activity, reduced motivation, and mental fatigue [23]. Each dimension contains 4 questions, with a range of scores between 4 and 20, generating a total score between 0 and 100. A higher score indicates more severe fatigue.

### 2.3. Dietary Assessment

Dietary data were assessed before study participants received any intervention or underwent any extensive physical investigations. The participants were instructed to maintain their regular diet during the recording days, and thus, the dietary data are believed to reflect participants’ habitual dietary intake. Participants were given oral and written instructions on how to perform the food record, which was recorded during four consecutive days (Wednesday–Saturday) in order to capture both weekdays and weekend days. All foods and drinks were reported and the quantity was estimated in grams or by using household utensils. Intake of energy, nutrients, and FODMAPs were calculated in a special version of the software Dietist XP 3.1 (Kostdata.se, Stockholm, Sweden), connected to an aggregated FODMAP database [24]. The food composition table was provided by the National Food Agency in Sweden. The FODMAP database contained information about fructose, fructan, lactose, galacto-oligosaccharide (GOS), and polyol content (g/100 g) from published sources [24]. A trained dietician entered all diet records into the software.

As fructose can be co-absorbed together with glucose [25,26], only fructose in excess of glucose counts as a FODMAP [10]. Excess fructose was calculated by subtracting intake of fructose (g)-glucose (g) for each separate meal. If the glucose content was higher than the fructose content, a value of 0 was denoted for excess fructose. Intakes of nutrients were first summarized for each meal, and thereafter summarized into intakes per day and finally presented as the mean intake of all four days. Cut offs for energy intake were set for energy levels ≤800 kcal/day or ≥4500 kcal/day in order to remove reports with implausible habitual intakes. No subjects exceeded these limits. For reported intake of FODMAPs, outliers were defined as those exceeding mean ± 4 SD, thus, and one subject was excluded. 

### 2.4. Statistical Analysis

Clinical characteristics’ and quantitative variables are presented as mean ± SD for women and men separately, and they were divided into subtypes of IBS, i.e., IBS-C, IBS-D, IBS-M, and IBS-U. Differences in means between the four IBS subtypes were analyzed by analysis of variance (ANOVA). As only 44 men took part in the study, only descriptive data are presented for men and correlation and regression analyses were performed in women only. We have previously demonstrated that FODMAP intake assessed using a four-day diet record has a limited precision [17]. Thus, for correlation and regression analyses, only rankings (i.e., quartiles) of energy-adjusted FODMAP intakes (g/1000 kcal) were used instead of absolute amounts. Spearman’s rank correlation was used for correlation analyses between quartiles of energy-adjusted FODMAP intake and questionnaire data assessing gastrointestinal and extraintestinal domains. To assess variables related to IBS symptom severity, linear regression analyses were used with IBS-SSS as a continuous dependent variable. Further, multivariable linear regression analyses were used to evaluate the associations between FODMAP intake (independent variable) and IBS-SSS (dependent variable). Two models were evaluated; one combining intake of all FODMAPs, and one model with total FODMAP intake without lactose. Subtypes of IBS were evaluated as an interaction variable. Variables evaluated for potential confounding effect included energy intake, dietary fiber, age, body mass index (BMI), GI-specific anxiety (VSI), somatic symptoms (PHQ), anxiety and depression (HAD), and fatigue (MFI-20). Variables that affected the beta coefficient with at least ± 10% were included in the final model. All statistical analyses were two-sided with a significance level at α < 0.05. Statistical analyses were performed using IBM SPSS Statistics for Windows, Version 22.0 (Armonk, NY, USA: IBM Corp.). 

## 3. Results

In total, 145 women and 44 men reported their food intake and completed all questionnaires. Participants’ characteristics can be seen in Table 1. The mean age was 37 years (median 33 and 32 years for women and men, respectively), and the majority of participants (60%) had a body mass index (BMI) within the normal range (i.e., BMI 20–24.9). Patients with IBS-U had less severe somatic symptoms (PHQ) compared with IBS-D and IBS-M. The severity of IBS symptoms did not differ significantly between subgroups of IBS.

### 3.1. Reported Diet Intake

For women, the distribution of macronutrients, expressed as percentage of energy intake, did not differ between subtypes of IBS. For men, a statistically significant higher intake of fat was reported in IBS-C, and reported alcohol intake was higher in IBS-D and IBS-M.

When comparing the reported FODMAP intakes among the different IBS subtypes, intakes appeared to be rather similar, Table 2. The only statistically significant difference was that intake of lactose was lower among women with IBS-D. A higher proportion of women with IBS-D, 17.5%, reported very low intakes of lactose (<2 g/day) compared to women with IBS-C (0%), IBS-M (0%), and IBS-U (5.6%). There was also a trend toward higher reported total FODMAP intake among women with IBS-M (*p* = 0.054). The variation in how frequently FODMAPs were consumed, expressed as coefficient of variation (CVw), did not differ significantly among IBS subtypes (data not shown).

### 3.2. Correlations between FODMAP Intake and Symptom Severity in Women

**Figure 1:**
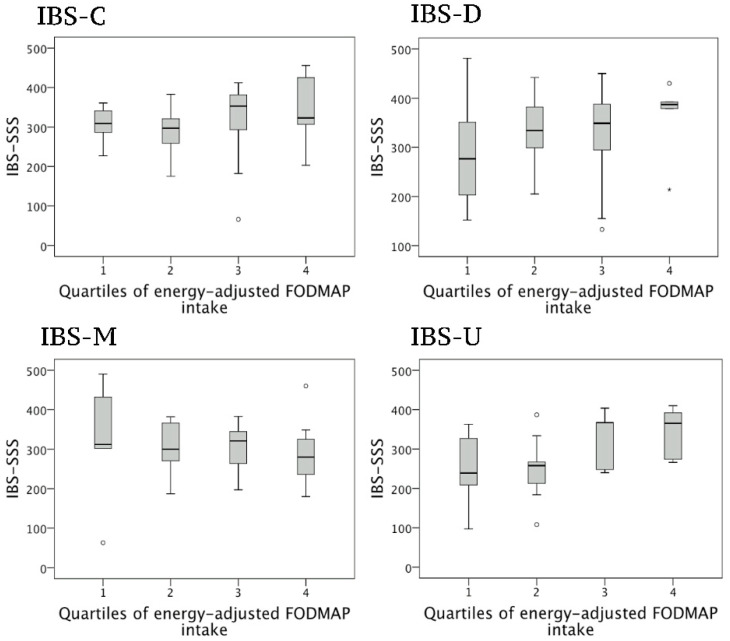
Boxplots are showing the relationship between quartiles of energy-adjusted intake of FODMAPs and IBS symptom severity among women within different subtypes of IBS. Box plots shows median values (solid horizontal line) 50th percentile values (box outline), 90th percentile values (whiskers), outlier values (circles) and extreme outliers (asterisks). Abbreviation: FODMAP, fermentable oligo-, di-, monosaccharides and polyols; IBS, irritable bowel syndrome; IBS-C, IBS with constipation, IBS-D, IBS with diarrhea, IBS-M, mixed IBS, IBS-U, unsubtyped IBS; IBS-SSS, IBS severity scoring system.

The relationship between reported intake of FODMAPs, using quartiles of energy-adjusted values, and IBS symptom severity, showed a weak but significant correlation when all women were combined (Table 3). The strongest correlation between FODMAP intake and IBS symptom severity was seen in the IBS-U subtype (Figure 1). Here, higher FODMAP intake correlated significantly to a higher pain frequency and daily life interference. Intake of FODMAP did not correlate to extraintestinal symptoms, fatigue, depression, or general or GI-specific anxiety (Table 3).

### 3.3. Univariable Regression Analyses

To further investigate how diet may impact on the severity of IBS symptoms, linear regression analyses were performed. Patient characteristics, reported dietary intake, and relationship with IBS symptom severity are presented in Table 4. IBS subtype was included as an interaction variable, but it was not statistically significant (*p* = 0.33).

Of all variables that were investigated, significant relationships were noted for energy intake, energy-adjusted FODMAP, and energy-adjusted excess fructose intake and IBS-SSS. For FODMAPs, these relationships were mainly driven by the subgroup IBS-U, where excess fructose intake accounted for 19.9% of the explained variance in symptom severity.

### 3.4. Multivariable Regression Analyses

Further, in multivariable models adjusting for energy intake, age, BMI, and somatic symptoms, the association between FODMAP intake and IBS symptom severity remained statistically significant in IBS-U and became statistically significant in IBS-M (Table 5). Interestingly, the slope points in the opposite direction for the two groups, indicating that increased FODMAP intake was associated with less symptoms in IBS-M, and with more symptoms in IBS-U (B = −28.42 and B = 21.89, respectively). As lactose accounts for approximately 50% of all FODMAPS, we also evaluated a second model where lactose was excluded from the sum of total FODMAPs. When lactose was excluded, the relationship between FODMAPs and symptom severity was no longer statistically significant in IBS-M.

## 4. Discussion

In this cross-sectional study, using a well-characterized group of patients with IBS with detailed dietary data, we studied how habitual FODMAP intake relates to symptom severity in different IBS subtypes based on the predominant bowel habit. Our study showed large similarities in FODMAP intake among patients with different IBS subtypes, even though minor differences were noted. Furthermore, weak associations between FODMAP intake and IBS symptom severity were demonstrated with differences among subtypes, where excess fructose intake accounted for a large share of explained variance in symptom severity among women with IBS-U. 

This study is one of few to link habitual FODMAP intake to symptom severity, and to study this relationship among different subtypes of IBS. A recent study published data on habitual FODMAP intake in a UK population, where the mean total FODMAP intake was 17 g/day [27], which is well in line with our findings. Another study performed in a Dutch setting has investigated the habitual diet of patients with IBS, and although the total FODMAP intake was not reported, the authors also found dissimilar results among the different subtypes of IBS [28]. For instance, a lower fiber intake correlated significantly with higher symptom scores among IBS-D, but this was not the case for the other subtypes. Furthermore, intake of apples significantly correlated to increased discomfort among IBS-C and IBS-M [28]. In that cohort, only 4.6% of the participants were classified as IBS-U, which might explain the lack of statistically significant findings in that subtype. 

In this study, reported intake of FODMAPs appeared quite similar among the different subtypes of IBS; the only significant difference was that women with IBS-D reported a lower daily lactose intake. This may be attributed to the higher proportion of women with IBS-D who appeared to exclude lactose from their diet. Lactose intolerance is a common condition among patients with IBS, as it is in the general adult population [29]. Symptoms of lactose intolerance may arise from lactose malabsorption, i.e., from the inability to produce lactase at the brush border, but it may also arise from an unfavorable composition of the intestinal microbiome or a history of GI disorders [29]. Symptoms of lactose intolerance include watery stools and bloating; hence, the symptoms of lactose intolerance overlap with symptoms of the diarrhea-predominant type of IBS. Notably, not all individuals that are lactose malabsorbers do experience symptoms after lactose intake, and on the other hand, individuals with visceral hypersensitivity may experience symptoms even at very low intakes [29]. Our study showed that lactose accounts for approximately 50% of the total FODMAP intake among patients with IBS, whereby intake of lactose has the potential to affect symptom generation to a large degree depending on if lactose is well tolerated or not. Whether lactose malabsorption is more prevalent among IBS-D than among other subtypes of IBS is unclear, but it is likely that patients in our study attributed their symptoms to intake of lactose and had therefore reduced their lactose intakes prior to the study measurement. 

Interestingly, we found no correlation between FODMAP intake and severity of bloating, which has been suggested as one of the main symptoms influenced by FODMAPs through bowel distension [30]. Again, this might be explained by a foregoing reduction in FODMAPs among individuals who experience FODMAP containing foods to cause bloating. 

We did not see any effect of CVw on symptom severity score, which indicates that individuals who report consuming regular amounts of FODMAPs experience the same grade of symptom severity as individuals who report a more irregular consumption. From this, we can conclude that regular FODMAP consumption is not clearly more beneficial over occasional intakes. 

There was a weak but statistically significant correlation between quartiles of energy-adjusted FODMAP intake and IBS symptom severity. This relationship was mainly driven by the subgroup IBS-U, where FODMAP intake correlated to a higher pain frequency and more bowel habit dissatisfaction. This was further studied in the regression models, and of all individual FODMAPs, excess fructose was clearly the most potent in generating symptoms. As much as 20% of all variance in IBS symptom severity was explained by excess fructose intake in IBS-U, which is remarkable. Fructose malabsorption is common both among healthy subjects as well as among individuals with functional GI disorders [31], and up to one-third of patients with IBS is thought to have insufficient fructose absorption [32]. Fructose is mainly absorbed in the small intestine by facilitated diffusion via glucose transporter (GLUT) 5 receptors, but it can also be absorbed by co-transport with glucose through GLUT2 receptors [25]. Symptoms of fructose malabsorption resemble those of lactose intolerance, i.e., increased flatulence and loose stools [33]. However, it is not apparent why patients with IBS-U in particular would have such increased symptom burden after ingestion of excess fructose. Whether fructose malabsorption is more common among IBS-U, or if the lack of abnormal stools makes the association between food intake and symptom generation more difficult to link, would be interesting for future research. 

In the regression analyses, we also demonstrated that reported energy intake was weakly, but statistically significantly, related to lower IBS symptom severity, meaning that individuals who reported a low intake of energy experienced having more GI symptoms than individuals who reported a higher energy intake. This could reflect that individuals who experience a lot of symptoms have reduced their food intake in an attempt to reduce pain. We have previously shown that the higher number of food items a person attributes to trigger symptoms, the more severe the IBS symptoms [34]. A qualitative study has described how women with IBS have developed “self-care strategies” to cope with their GI symptoms, which include a “trial and error” method to exclude foods believed to trigger symptoms, and to reintroduce them again if the symptoms did not improve [35]. Consequently, patients experiencing a heavy IBS symptom burden may exclude more foods from their diet, leading to a lower energy intake. 

In the multivariable regression model, when FODMAP intake was adjusted for energy intake, age, BMI, and other somatic symptoms, the final full model remained statistically significant for IBS-U and also became statistically significant for IBS-M. However, women with IBS-M had less symptoms with increasing FODMAP intake, whereas women with IBS-U had increasing symptom burden with higher intake. From the bivariate regression models, one can note that intake of lactose (although not statistically significant) seems to contribute to less severe symptoms in IBS-M, and that the reported lactose intake was highest in this group. In the second full model, where lactose was removed from the total sum of FODMAPs, symptom severity was no longer related to FODMAP intake in IBS-M. One can assume that this reflects that lactose absorbers have continued to consume lactose and, when tolerable, lactose does not yield negative effects on symptom severity. 

A strength of this study was the inclusion of a large number of well-characterized IBS patients, covering a wide range of IBS symptoms. However, there were too few male participants in this study to be able to perform correlation or regression analyses with sufficient power in women and men separately; thus, these analyses were performed on women only. As participants were recruited both at regular outpatient clinics as well as through advertisement in the local newspaper, the study participants likely represent an IBS population where the results can be generalizable. The use of validated and well-established questionnaires to characterize participants and symptoms is also a key strength. On the negative side is the lack of longitudinal data; as we only have access to one measurement of food intake and symptom severity taken concurrently, we do not know whether patients have already changed their diet in order to manage symptoms. Therefore, we cannot rule out reversed causality, and the results must be interpreted with caution regarding cause and effect.

In summary, the intake of FODMAPs seems to exert varying effects in individuals with IBS, and there might be some similar traits within patients with the same subtype of IBS. As the different types of FODMAPs seem to be more or less potent in generating symptoms, it is warranted to study the effect of each FODMAP separately and among the different subtypes of IBS, in longitudinal studies or randomized controlled trials. 

## 5. Conclusions

The reported intake of FODMAPs was quite similar among IBS subtypes based on the predominant bowel habit, except for a lower intake of lactose among women with IBS-D. Intake of FODMAPs was related to more severe IBS symptoms with differences among IBS subtypes, and this relationship was driven mainly by women with IBS-U. Here, excess fructose intake was related to increased symptom severity. However, patients who are intolerant to certain FODMAPs may already have reduced their FODMAP intake, and therefore, we cannot rule out reversed causality.

## Figures and Tables

**Table 1:** Demographic and clinical data among all women and men with irritable bowel syndrome participating in the study.

	IBS-C	IBS-D	IBS-M	IBS-U	Difference between Groups
	Mean ± SD	Mean ± SD	Mean ± SD	Mean ± SD	*p*-Value
**Women (*n* = 145)**	(*n* = 36)	(*n* = 40)	(*n* = 33)	(*n* = 36)	
Age, y	40.2 ± 15.6	35.6 ± 13.3	35.0 ± 13.2	39.7 ± 16.1	0.30
Weight, kg	65 ± 11.6	65 ± 12.7	65.7 ± 16.1	63.0 ± 9.1	0.84
BMI (kg/m^2^)	23.0 ± 3.3	23.6 ± 4.3	23.4 ± 4.7	22.5 ± 2.8	0.68
**Men (*n* = 44)**	(*n* = 8)	(*n* = 14)	(*n* = 12)	(*n* = 10)	
Age, y	40.5 ± 11.8	39.7 ± 16.0	34.1 ± 13.5	35.5 ± 12.3	0.65
Weight, kg	78.6 ± 11.3	80.5 ± 10.4	81.8 ± 9.6	85.4 ± 15.8	0.68
BMI (kg/m^2^)	23.3 ± 3.6	24.5 ± 3.5	25.2 ± 2.1	25.5 ± 4.3	0.52
**All (*n* = 189)**	(*n* = 44)	(*n* = 54)	(*n* = 45)	(*n* = 46)	
IBS-SSS	306 ± 81	302 ± 92	304 ± 84	291 ± 83	0.83
Pain intensity	52 ± 26	48 ± 27	54 ± 25	46 ± 24	0.48
Pain frequency	58 ± 34	52 ± 35	53 ± 33	54 ± 32	0.81
Bloating severity	61 ± 29	58 ± 27	58 ± 31	60 ± 28	0.92
Bowel habit dissatisfaction	65 ± 28	72 ± 23	73 ± 21	61 ± 29	0.065
Daily life interference	70 ± 23	72 ± 18	66 ± 26	69 ± 24	0.61
VSI	48.5 ± 17.5	43.7 ± 16.1	41.9 ± 15.9	48.1 ± 14.4	0.14
PHQ	12.2 ± 4.0	13.8 ± 5.6	14.3 ± 4.7	11.6 ± 4.3	0.025 *
HAD	12.9 ± 8.1	14.2 ± 6.2	13.6 ± 7.0	12.1 ± 7.4	0.53
MFI-20	59.1 ± 15.4	62.0 ± 16.2	60.7 ± 17.3	55.9 ± 16.2	0.32

Abbreviations: BMI, body mass index; IBS, irritable bowel syndrome; VSI, visceral sensitivity index; HAD, Hospital Anxiety and Depression Scale; PHQ, The Patient Health Questionnaire; MFI-20, Multidimensional Fatigue Inventory-20; IBS-C, IBS with constipation; IBS-D, IBS with diarrhea; IBS-M, mixed type IBS; IBS-U, unsubtyped IBS; IBS-SSS, IBS severity scoring system. * *p*-values < 0.05.

**Table 2:** Average reported daily energy, macronutrient, dietary fiber, and FODMAP intake among women and men with irritable bowel syndrome, divided into subtypes of IBS.

	IBS-C	IBS-D	IBS-M	IBS-U	Difference between Groups
	Mean ± SD	Mean ± SD	Mean ± SD	Mean ± SD	*p*-Value
**Women (*n* = 145)**	(*n* = 36)	(*n* = 40)	(*n* = 33)	(*n* = 36)	
Energy, kcal	1943 ± 487	1982 ± 497	2076 ± 546	2132 ± 462	0.36
Protein, E%	17.4 ± 3.2	16.4 ± 3.4	16.1 ± 3.6	15.4 ± 2.7	0.087
Carbohydrates, E%	38.0 ± 8.5	40.8 ± 6.6	41.3 ± 9.8	40.8 ± 6.9	0.31
Fat, E%	38.7 ± 8.5	38.2 ± 6.1	38.2 ± 8.5	38.5 ± 6.1	0.99
Alcohol, E%	4.0 ± 4.6	2.3 ± 2.9	2.4 ± 3.2	3.5 ± 2.7	0.10
Dietary fiber, E%	1.8 ± 0.6	2.1 ± 0.8	1.9 ± 0.5	1.9 ± 0.7	0.39
Dietary fiber, g	17.8 ± 7.4	20.6 ± 8.8	19.6 ± 7.0	20.0 ± 7.0	0.43
Total FODMAPs, g	19.0 ± 8.2	17.0 ± 8.3	23.9 ± 13.2	20.1 ± 12.1	0.054
Galacto-oligosaccharides, g	0.5 ± 0.4	0.5 ± 0.4	0.6 ± 0.6	0.4 ± 0.2	0.25
Fructans, g	2.0 ± 0.9	2.3 ± 1.2	2.8 ± 1.6	2.5 ± 1.1	0.08
Polyols, g	1.4 ± 1.6	0.9 ± 1.3	1.5 ± 1.7	0.9 ± 1.0	0.14
Lactose, g	10.0 ± 6.2	7.6 ± 6.6	13.6 ± 8.1	10.0 ± 8.3	0.009 *
Excess fructose, g	5.1 ± 3.7	5.6 ± 5.7	5.4 ± 7.8	6.3 ± 7.2	0.87
**Men (*n* = 44)**	(*n* = 8)	(*n* = 14)	(*n* = 12)	(*n* = 10)	
Energy, kcal	2292 ± 354	2309 ± 515	2432 ± 710	2369 ± 516	0.93
Protein, E%	16.9 ± 1.4	15.1 ± 2.4	18.4 ± 5.1	19.0 ± 8.2	0.21
Carbohydrates, E%	38.6 ± 8.8	44.5 ± 6.4	37.6 ± 8.4	43.0 ± 5.0	0.065
Fat, E%	42.1 ± 6.9	32.0 ± 6.8	36.7 ± 8.8	34.5 ± 7.9	0.037 *
Alcohol, E%	0.9 ± 1.7	6.1 ± 6.0	5.7 ± 5.4	1.5 ± 2.0	0.017 *
Dietary fiber, E%	1.5 ± 0.4	2.0 ± 0.7	1.6 ± 0.3	1.9 ± 0.8	0.182
Dietary fiber, g	18.1 ± 6.7	23.2 ± 9.3	18.5 ± 5.5	21.4 ± 8.3	0.343
Total FODMAPs, g	19.9 ± 9.1	19.6 ± 12.2	21.7 ± 18.4	28.9 ± 12.0	0.38
Galacto-oligosaccharides, g	0.5 ± 0.2	0.8 ± 0.6	0.5 ± 0.6	0.6 ± 0.5	0.52
Fructans, g	2.3 ± 1.2	3.2 ± 2.1	2.4 ± 1.0	3.3 ± 1.9	0.40
Polyols, g	1.0 ± 1.5	0.5 ± 0.7	0.8 ± 1.4	1.7 ± 2.5	0.36
Lactose, g	11.1 ± 7.8	9.5 ± 8.6	11.1 ± 10.5	14.5 ± 12.6	0.69
Excess fructose, g	5.1 ± 5.0	5.6 ± 4.4	6.9 ± 11.8	8.8 ± 5.1	0.69
**All (*n* = 189)**	(*n* = 44)	(*n* = 54)	(*n* = 45)	(*n* = 46)	
Total FODMAPs, g/1000 kcal	9.73 ± 3.96	8.83 ± 4.91	10.90 ± 5.99	10.13 ± 5.57	0.25
Galacto-oligosaccharides, g/1000 kcal	0.24 ± 0.21	0.30 ± 0.30	0.27 ± 0.31	0.21 ± 0.15	0.27
Fructans, g/1000 kcal	1.03 ± 0.41	1.24 ± 0.67	1.24 ± 0.60	1.24 ± 0.62	0.28
Polyols, g/1000 kcal	0.70 ± 0.83	0.41 ± 0.60	0.60 ± 0.69	0.50 ± 0.72	0.23
Lactose, g/1000 kcal	5.15 ± 3.02	4.18 ± 4.26	6.14 ± 3.94	5.00 ± 4.20	0.11
Excess fructose, g/1000 kcal	2.59 ± 1.98	2.69 ± 2.33	2.64 ± 3.72	3.18 ± 3.11	0.75

Abbreviations: E%, percentage of energy; FODMAPs, fermentable oligo-, di-, monosaccharides and polyols; IBS-C, IBS with constipation; IBS-D, IBS with diarrhea; IBS-M, mixed type IBS; IBS-U, unsubtyped IBS. * *p*-values < 0.05.

**Table 3:** Spearman correlation between quartiles of (energy adjusted) total FODMAP intake and IBS symptom severity and other symptoms among women divided into different IBS subtypes.

	All	IBS-C	IBS-D	IBS-M	IBS-U
IBS-SSS total	0.217 **	0.246	0.257	−0.159	0.518 **
Pain intensity	0.153	0.276	−0.069	0.088	0.258
Pain frequency	0.168 *	0.081	0.220	−0.061	0.386 *
Bloating severity	0.104	0.232	0.214	−0.229	0.188
Bowel habit dissatisfaction	0.124	0.090	0.088	−0.160	0.323
Daily life interference	0.159	0.229	0.086	0.090	0.347 *
VSI	−0.058	−0.075	0.100	−0.120	−0.015
PHQ	0.091	0.175	0.148	0.059	0.090
HAD	0.095	0.278	−0.036	0.148	0.047
MFI-20	0.046	0.132	0.010	0.037	−0.014

Abbreviations: IBS-SSS, irritable bowel severity scoring system; IBS-D, IBS with diarrhea; IBS-C, IBS with constipation; IBS-M, mixed IBS; IBS-U, unsubtyped IBS. * Correlation is significant at *p* < 0.05, ** correlation is significant at *p* < 0.01.

**Table 4:** Univariable linear regression investigating associations between age, BMI and dietary components and IBS symptom severity in women and divided into IBS subtypes.

Independent Variable	All			IBS-C			IBS-D			IBS-M			IBS-U		
	R^2^	B	*p*-Value	R^2^	B	*p*-Value	R^2^	B	*p*-Value	R^2^	B	*p*-Value	R^2^	B	*p*-Value
Age	0.009	0.545	0.208	0.051	−1.226	0.141	0.003	0.363	0.692	0.000	−0.022	0.982	0.049	−1.199	0.141
BMI	0.001	−0.633	0.709	0.021	−3.664	0.368	0.000	−0.396	0.902	0.023	−2.949	0.340	0.046	5.128	0.174
Energy, kcal	0.029	−0.030	0.042 *	0.009	−0.033	0.261	−0.022	−0.012	0.698	0.012	−0.035	0.249	−0.001	−0.031	0.330
CVw kcal	0.001	0.242	0.683	0.036	1.178	0.266	0.030	−1.246	0.283	0.023	−1.114	0.404	0.104	2.374	0.055
CVw FODMAP	0.003	−0.199	0.532	0.017	−0.654	0.447	0.023	−0.569	0.355	0.066	−0.992	0.156	0.072	0.846	0.115
EA FODMAP	0.043	16.32	0.012 *	0.039	15.09	0.250	0.046	18.89	0.186	0.009	−7.735	0.607	0.233	33.77	0.003 *
EA GOS	0.000	−1,154	0.859	0.052	−16.19	0.182	0.007	−6.438	0.605	0.000	−1.246	0.933	0.047	18.38	0.204
EA FOS	0.000	0.068	0.992	0.013	−8.814	0.514	0.005	−5.303	0.679	0.009	7.296	0.602	0.019	10.71	0.420
EA polyols	0.001	2.238	0.731	0.000	1.034	0.938	0.003	−4.255	0.745	0.022	10.09	0.415	0.000	0.101	0.995
EA lactose	0.000	−0.606	0.927	0.009	−8.583	0.589	0.006	−6.460	0.629	0.028	−14.06	0.370	0.070	18.44	0.124
EA excess fructose	0.045	16.09	0.011 *	0.029	12.49	0.318	0.042	15.33	0.213	0.000	−0.071	0.996	0.199	34.52	0.007 *

Abbreviations: CVw, coefficient of variation within individuals; EA, energy adjusted values g/1000 kcal; IBS, irritable bowel syndrome; IBS-C, IBS with constipation; IBS-D, IBS with diarrhea; IBS-M, mixed IBS; IBS-U, unsubtyped IBS; IBS-SSS, IBS severity scoring system * *p*-values <0.05.

**Table 5:** Multivariable linear regression for women with IBS studying the associations between FODMAP intake (energy-adjusted, quartiles) and IBS symptom severity.

	All			IBS-C			IBS-D			IBS-M			IBS-U		
	Adjusted R^2^	B	*p*-Value	Adjusted R^2^	B	*p*-Value	Adjusted R^2^	B	*p*-Value	Adjusted R^2^	B	*p*-Value	Adjusted R^2^	B	*p*-Value
**Full model 1** **EA FODMAP**	**0.246**	**5.092**	**0.362**	**0.338**	**7.752**	**0.523**	**0.174**	**14.82**	**0.251**	**0.273**	**−28.42**	**0.023**	**0.313**	**21.89**	**0.047**
Energy intake		−0.013	0.362		−0.010	0.696		0.004	0.882		−0.016	0.526		−0.026	0.286
Age		0.116	0.791		−0.104	0.910		1.434	0.161		−0.333	0.712		−0.853	0.259
BMI		−2.668	0.106		−4.163	0.320		−1.945	0.528		−3.692	0.192		−0.151	0.976
PHQ		8.217	0.000		13.16	0.001		7.784	0.007		8.457	0.008		5.938	0.040
**Full model 2** **EA FODMAP minus lactose**	**0.241**	**1.860**	**0.744**	**0.327**	**−1.676**	**0.886**	**0.137**	**−1.873**	**0.876**	**0.123**	**−12.15**	**0.386**	**0.336**	**26.10**	**0.029**
Energy intake		−0.014	0.243		−0.014	0.607		0.000	0.988		−0.005	0.860		−0.023	0.349
Age		0.142	0.746		−0.008	0.993		1.545	0.139		−0.240	0.810		−0.993	0.191
BMI		−2.685	0.111		−4.246	0.330		−1.851	0.567		−3.350	0.277		−1.055	0.823
PHQ		8.243	0.001		13.49	0.001		8.343	0.005		9.118	0.014		7.212	0.010

Abbreviations: EA, energy adjusted values g/1000 kcal; IBS, irritable bowel syndrome; IBS-C, IBS with constipation; IBS-D, IBS with diarrhea; IBS-M, mixed IBS; IBS-U, unsubtyped IBS; IBS-SSS, IBS severity scoring system.

## Data Availability

Data is available on request due to ethical restrictions.
